# Electroacupuncture therapy and bone cancer pain relief: experimental study on analgesic mechanisms in rats

**DOI:** 10.3389/fpain.2025.1597472

**Published:** 2025-10-03

**Authors:** Yanhua Li, Fangfei Li, Caizhi Xiao, Jie Cao, Dongqin Xia, Yongzhong Wu

**Affiliations:** Chongqing Key Laboratory of Translational Research for Cancer Metastasis and Individualized Treatment, Chongqing University Cancer Hospital, Chongqing, China

**Keywords:** bone cancer pain, electroacupuncture, astrocytes, neuroimmune inflammation, analgesia

## Abstract

**Background:**

Bone cancer pain is a common complication of advanced malignant tumors.Chemotherapeutic drugs, regardless of their origin or type, are often associated with various adverse effects such as gastrointestinal toxicity, immune suppression, and acquired drug resistance, which can compromise patients’ quality of life and treatment compliance. Electroacupuncture, known for its safety and analgesic effects, has been increasingly studied in bone cancer pain but the underlying mechanism is not fully understood.

**Objective:**

To explore the mechanism of electroacupuncture in rats with bone cancer pain.

**Methods:**

Forty-eight SD rats were divided into four groups: blank control, sham electroacupuncture, electroacupuncture-1, and electroacupuncture-2, with 12 rats in each group. Except the control group, rats were inoculated with cancer cells in the left tibia to induce bone cancer pain. The electroacupuncture groups received interventions starting on the 6th day after modeling. Mechanical pain sensitivity (PWT) and thermal pain sensitivity (PWL) were assessed, and the expression of phosphorylated glycogen synthase kinase-3 (p-GSK-3) and glial fibrillary acidic protein (GFAP) in the spinal cord were analyzed. HE staining was used to observe tibial pathological changes.

**Results:**

From the 6th day, PWT and PWL were significantly reduced in the control group compared to the sham group (*P* < 0.05). Electroacupuncture-1 significantly increased PWT and PWL compared to the sham group (*P* < 0.05), while no significant changes were observed in electroacupuncture-2 compared to the control. On day 12, spinal p-GSK-3 levels were significantly lower and GFAP levels significantly higher in the model and control groups compared to the electroacupuncture-1 group (*P* < 0.05). The electroacupuncture-2 group showed no significant changes. Inflammatory cytokines IL-1, IL-6, and TNF-α were significantly elevated in the model group compared to the control (*P* < 0.05), but significantly reduced in the electroacupuncture-1 group (*P* < 0.05). HE staining showed cancer cell infiltration and bone tissue damage in the sham and electroacupuncture groups.

**Conclusion:**

Electroacupuncture significantly reduced the pain threshold in rats with bone cancer pain. This effect is likely due to the down-regulation of GSK-3 activity, inhibition of astrocyte activation, and reduction in inflammatory responses.

## 1 Introduction

Bone cancer pain (BCP) is a severe and frequent complication in patients with advanced malignancies, particularly those with bone metastases (1). Tumor invasion into the bone matrix causes structural destruction, nerve compression, and release of inflammatory mediators, resulting in persistent nociceptive hypersensitivity and reduced quality of life. While several animal models have been established to replicate cancer-induced bone pain, the most commonly used involve localized tumor cell inoculation into the tibia, which mimics focal bone lesions rather than systemic metastatic disease (2, 3). Despite this limitation, such models are widely accepted for evaluating the pathophysiology of BCP and testing therapeutic strategies including non-pharmacologic interventions such as electroacupuncture (EA) (4). Therefore, exploring the mechanism of bone cancer pain and developing new effective therapeutic agents are important directions in the current treatment of malignant tumours. Acupuncture analgesia, as a widely recognised Chinese medicine therapy, has been shown to be effective in pain relief (4). A large number of studies have shown that acupuncture has certain practicality and safety in the treatment of cancer pain (5). Electroacupuncture, which combines traditional acupuncture with neuroelectric stimulation and is based on the theory of traditional Chinese medicine, has been widely used in the treatment of pain-related diseases, and has been widely applied in clinical practice because it is safe, effective and has few side effects (6), and analgesic treatment based on the identification of acupoints for immediate analgesia has been shown to be effective (7). Acupuncture analgesia involves a complex network of regulatory mechanisms from the periphery to the central nervous system, containing the participation of multiple molecular pathways, inflammatory factors, neurotransmitters, and acupoint properties, but the specific mechanisms remain unclear (8). The control of cancer pain in the middle and late stages is crucial to the quality of life of patients, and the study of the mechanism of acupuncture in the treatment of cancer pain can help to better apply the method to improve the prognosis of patients in the clinic. To date, studies on the mechanism of electroacupuncture against bone cancer pain are more limited. Glycogen synthase kinase-3β (GSK-3β) is a key point in the convergence of multiple signalling pathways and may be involved in the development of chronic pain (9). Therefore, this study investigates the potential effects and mechanisms of electroacupuncture.

## 2 Materials and methods

### 2.1 Laboratory animal

Clean-grade healthy female SD rats [Animal Production Licence No.: SCXK (Shanghai) 2018-0006] weighing 160–180 g were selected and purchased from our animal laboratory. Room temperature was maintained at 21–25℃, humidity was maintained at 40%–60%, rats were free to ingest and drink water, and 12 h cyclic light exposure. All the operation procedures of this experiment were approved by the Animal Ethics Committee of the hospital. All patients were divided into blank control group, sham electroacupuncture group, electroacupuncture-1 group, electroacupuncture-2 group, 12 rats in each group by using the random number table method after acclimatisation feeding for 7d.

### 2.2 Main reagents and instruments

GFAP, p-GSK-3β, GSK-3β antibody (Abcam, UK), IL-1β, IL-6, TNF-α, ELISA kit (Shanghai Enzyme-linked Biologicals), Fluo ViewFV1200 laser scanning confocal microscope (Olympus, Japan).

## 3 Methodology

### 3.1 Moulding

Bone cancer pain model: modelling by cancer cell inoculation. MRMT-1 breast cancer cells preserved in liquid nitrogen were taken out, resuscitated and cultured, and then placed in medium containing 1% penicillin-streptomycin solution, 10% fetal bovine serum, 89% RPMI 1,640, and cultured in a 5% carbon dioxide incubator at 37 °C for 7 d. On the day of the modelling, the cells growing on the wall of the culture flasks were washed with PBS, and then digested with 0.25% trypsin for 20 s, and the cell suspension was collected by centrifugation at 1,200 r/min for 3 min at room temperature, and the cell suspension was configured as 1 × 10 using a cell counter. On the day of modelling, the cells were washed with PBS, digested with 0.25% trypsin for 20 s, centrifuged at 1,200 r/min for 3 min at room temperature, and the cell suspension was collected, configured with a concentration of 1 × 10^7^ cells/ml using a cell counter, and placed in an ice box at 4 °C for spare use. The rats were anaesthetized by low-flow inhalation anesthesia with isoflurane (output concentration of 2%, oxygen concentration of 500 ml/min). After successful anesthesia, the rats were placed in the supine position, and after disinfection and preparation of the skin of the left tibia, an incision of about 1 cm in length was made along the upper section of the tibial spine to fully expose the tibial head. At the tibial tuberosity, a 9-gauge needle was used to drill a hole into the medial side of the tibia at an angle of 30–45°, and 3 μl of MRMT-1 breast cancer cell suspension (about 1 × 10^4^ cancer cells) was injected into the marrow cavity of the tibia by using a micro syringe, and the needle was withdrawn after a 1-min stay, and the hole in the bone was quickly sealed with sterile bone wax, and the wound was washed with 0.9% sterile sodium chloride solution and sutured, and finally 0.2 ml of sodium benzylpenicillin was injected into the wound intramuscularly to prevent infection. penicillin sodium to prevent infection. A significant decrease in mechanical paw withdrawal threshold (PWT) 10 d after cancer cell inoculation was taken as the success criterion for bone cancer pain modelling. In the blank control group, the same dose of sterile PBS was injected into the bone marrow cavity of the left tibia, and the rest of the operation was the same as that of the bone cancer pain group.

### 3.2 Methods of intervention

In order to ensure that the animals were in a stable and controllable state before electroacupuncture treatment and to avoid stress reactions, the experiment was conducted using homemade fixation sets to treat all groups of animals, including the control group. After the animals were quiet, the electroacupuncture experiment was carried out. The sham electro-acupuncture group was needled bilaterally with 5 mm subcutaneous needling next to the foot Sanli point and Kunlun point without electricity. The electroacupuncture-1 group was given electroacupuncture intervention starting at the 6th d after rat modelling, selecting bilateral foot Sanli and Kunlun points, and giving sparse and dense waves 2/100 Hz, intensity 0.5–1.5 mA, each point for 30 min each time, every other day, for a total of 7 times. The electroacupuncture-2 group started to give electroacupuncture intervention on the 6th d after rat modelling, selecting bilateral foot Sanli points and Kunlun points, and giving sparse and dense waves 2/100 Hz, of which 1 mA for 15 min and 2 mA for 15 min, each point for 30 min each time, once every other day, for a total of 7 times.

### 3.3 Behavioural tests for altered pain behaviour in rats

The mechanical pain sensitivity of rats was expressed as the Paw withdrawal threshold (PWT). The rats were placed in a cage with wire mesh at the bottom and Plexiglas at the periphery, and the left plantar foot of the rats was stimulated with a fibre wire with a diameter of about 0.5 mm (StoeIting Company, USA) to avoid the foot pads, and the rats showed a withdrawal reflex, which was the pressure value of PWT. Measurements were taken 5 times consecutively, with an interval of 3–5 min each time, and the average value was obtained by removing the maximum and minimum values. The thermal pain sensitivity of the rats was detected using a thermal pain stimulator, expressed as (Paw withdrawal latcncy, PWL), and the heat intensity was set at 16 s latency of the heat-shrinking foot, and the time of avoidance of the foot-shrinking reflex of the rats using the thermal pain stimulator to stimulate the skin in the middle of the left plantar surface of the rats was defined as the PWL (s).

### 3.4 Immunofluorescence assay for GFAP expression in rat spinal cord

Rats were executed by decapitation after anaesthesia and perfused with 4% paraformaldehyde solution to fix the tissues. The tissues were cut into 30 μ m thick sections and rinsed thoroughly. On d 1, the sections were incubated for 1 h at room temperature with the addition of GFAP primary antibody (dilution ratio 1:500), and then overnight in a 4 °C environment. On the 2nd d, after rinsing the sections, fluorescent secondary antibody was added and incubated away from light for 2 h. After sealing, the sections were stained and observed using a laser confocal microscope and photographs were taken to record the results.

### 3.5 Detection of protein expression of GFAP, p-GSK-3 in rat spinal cord by protein blotting method

Rats were executed by decapitation after anaesthesia and perfused with 4% paraformaldehyde solution to fix the tissues. The tissues were cut into 30 μ m thick sections and rinsed thoroughly. Protein quantification was performed with reference to the instructions of the Diquinoline Carboxylic Acid Protein Quantification Kit, and the unfolded sodium dodecyl sulfate-polyacrylamide gel electrophoresis was completed with membrane transfer. The specific operation procedure was referred to the literature (10). Enhanced chemiluminescence was performed by dropping a chemiluminescent solution into it, and the relative expression of the bands was derived by grey scale quantitative analysis after exposure imaging.

### 3.6 Determination of Il-1β, Il-6 and TNF-α in rat spinal cord by ELISA

Rats were executed by decapitation after anaesthesia and perfused with 4% paraformaldehyde solution to fix the tissues (11). The tissues were cut into 30 μ m thick sections and rinsed well. The levels of IL-1β, IL-6 and TNF-α in the spinal cord were measured by ELISA according to the reagent instructions.

### 3.7 Pathological testing of the tibia

After the last intervention was completed, the rats were executed and the ligaments, skin and muscles between their tibia and femur and metatarsal bones were cut. After the muscles and other tissues on the surface of the tibia were separated using a scalpel, they were rinsed clean using saline. The tibia was then fixed in 4% paraformaldehyde buffer for 24 h, followed by immersion in decalcification solution at a volume ratio of 1:10 for 36 h. The upper 30% portion of the tibia proximal to the end of the femur was cut using surgical scissors, and rinsed with distilled water, followed by two rinses with 50% alcohol. Subsequently, a concentration gradient of 70%, 80%, 95% and 100% alcohol was sequentially used for dehydration, followed by xylene for hyalinisation. After that, the paraffin was embedded with paraffin wax and cut into 4 μ m thick paraffin slices using a slicer. These paraffin slices were unfolded in water at 50 °C and dried in a constant temperature oven at 63 °C for 2 h. After cooling, the slices were placed under a light microscope for observation and photographed for documentation.

### 3.8 Peripheral blood collection and monocyte count analysis

Peripheral blood samples were collected from all rats on day 15 after behavioral testing. Approximately 1.0 ml of blood was obtained via abdominal aorta puncture under anesthesia and transferred into EDTA anticoagulant tubes. Total and differential white blood cell counts were analyzed using an automated hematology analyzer (Mindray BC-2800Vet, China), with specific attention to absolute monocyte counts (expressed in 10^9^/L). Eight animals per group were included in the analysis.

### 3.9 Immunofluorescence co-localization of tumor-infiltrating immune markers and astrocytic/p-GSK-3β in the spinal cord

To assess the spatial co-expression of tumor-infiltrating immune cell markers and astrocyte/p-GSK-3 signaling in the spinal cord, immunofluorescence double-label staining was performed on transverse sections from all experimental groups. After final behavioral assessments, rats were deeply anesthetized and perfused with cold phosphate-buffered saline (PBS), followed by 4% paraformaldehyde (PFA). Lumbar spinal cord segments (L4–L6) were harvested, post-fixed in 4% PFA for 24 h at 4 °C, and cryoprotected in 30% sucrose until fully equilibrated.Tissue was embedded in OCT compound and sectioned into 30 μm transverse slices using a cryostat (Leica CM1950). Sections were mounted on poly-L-lysine-coated slides and air-dried. After blocking with 5% bovine serum albumin (BSA) for 1 h at room temperature, primary antibody pairs were applied and incubated overnight at 4 °C in a humidified chamber. The following marker combinations were used:CD68 (macrophage marker, red channel) + GFAP (astrocyte marker, green channel),Iba1 (microglia marker, red) + p-GSK-3 (green),CD11b (microglia/macrophage marker, red) + GFAP (green).After washing with PBS, appropriate secondary antibodies conjugated to Alexa Fluor® 488 or 594 (1:500, Thermo Fisher Scientific) were applied for 1 h at room temperature in the dark. Nuclei were counterstained with DAPI (1 μg/ml) for 5 min. After final washes, sections were mounted using antifade medium and sealed.Images were captured using a laser scanning confocal microscope (FV1200, Olympus, Japan) under a 40× oil immersion objective. A standardized scale bar of 30 μm was applied to all representative merged images. Co-localization was analyzed using ImageJ (NIH, USA), and Pearson's correlation coefficients were calculated for quantitative assessment (*n* = 4 animals per group, three fields per section).

### 3.10 Bone matrix and osteoclast activity assessment

After intervention, proximal tibial sections were subjected to immunohistochemical staining to detect bone matrix proteins (osteocalcin, collagen I) and osteoclast markers (TRAP, RANKL). Following decalcification, 5 µm paraffin sections were prepared, antigen retrieval performed, and primary antibodies applied overnight at 4 °C. Staining intensity was semi-quantitatively scored and compared across groups.

### 3.11 Statistical treatment

Graphpad Prism 8.0 was applied for data plotting and statistical analysis, and the experimental data were expressed as mean ± standard error ($\bar{x}\pm s$) were used to express the experimental data. Data between two groups were analysed by Student's *t*-test, and comparisons between multiple groups were made by one-way ANOVA, and *P* < 0.05 was taken as statistically significant difference.Prior to parametric analyses, all data were tested for normal distribution using the Shapiro–Wilk test. Variables with *P* > 0.05 were considered to follow a normal distribution and thus suitable for parametric testing.

## 4 Results

### 4.1 Comparison of the expression levels of PWT and PWL in rats of each group

**Figure 1:**
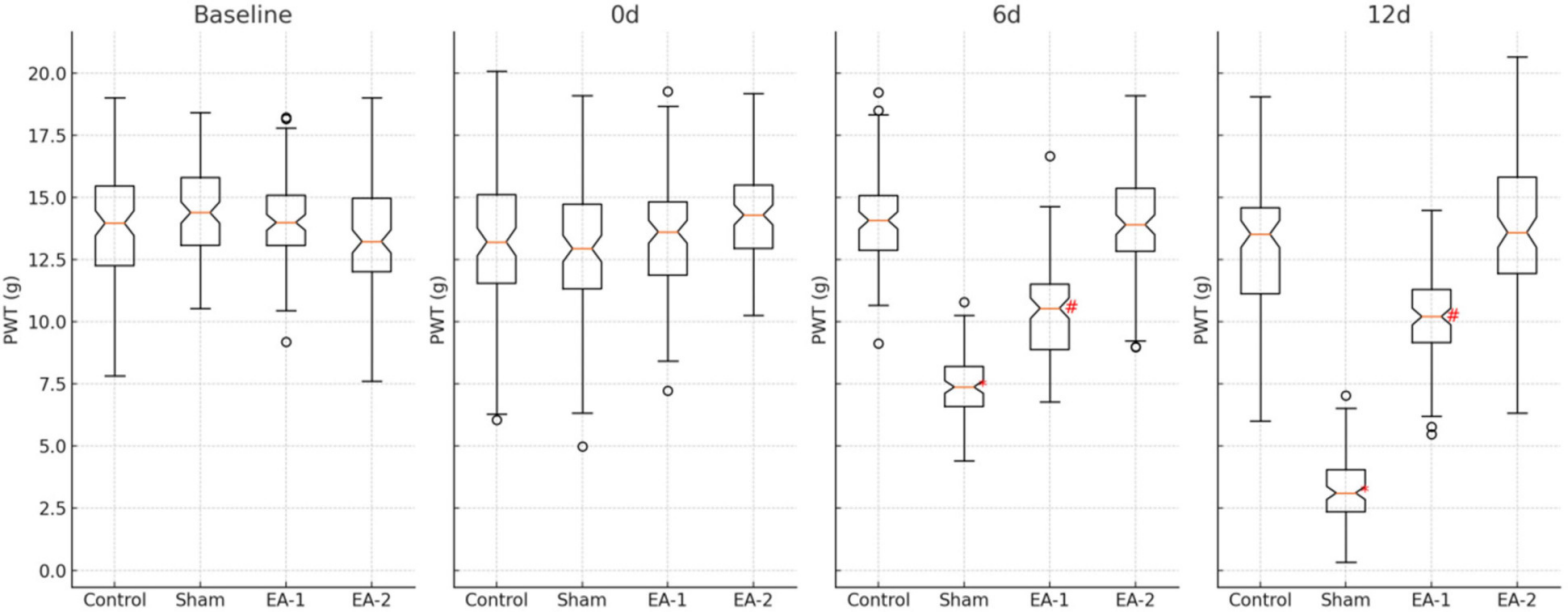
Effect of electroacupuncture on mechanical foot threshold (PWT) in rats.

**Figure 2:**
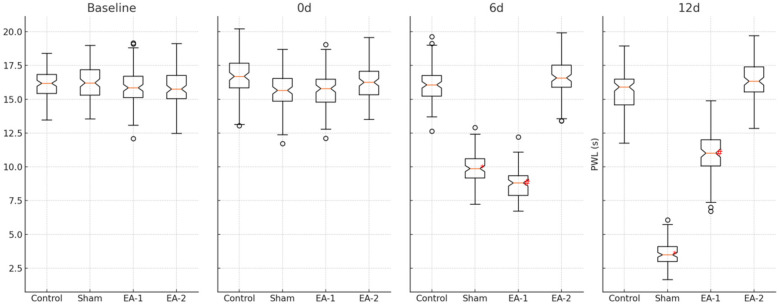
Effect of electroacupuncture on latency of heat shrinkage group (PWL) in rats.

From the 6th d onwards, the expression levels of PWT and PWL were significantly reduced in the blank control group compared with the sham electroacupuncture group, and the differences were statistically significant (all *P* < 0.05), the levels of PWT and PWL were significantly increased in the electroacupuncture-1 group compared with the sham electroacupuncture group, and the differences were statistically significant (all *P* < 0.05), and the levels of PWT and PWL were not statistically significant (all *P* *>* 0.05), when comparing the electroacupuncture-2 group with the blank control group, the differences were not statistically significant (see Table 1, Figure 2). PWL levels were all statistically insignificant (*P* > 0.05), see Table 1, Figure 1, Table 2, Figure 2.

**Table 1:** Effect of electroacupuncture on mechanical foot threshold (PWT) in rats ($\bar{x}\pm s$).

Group	n	PWT (g)			
		base line (in geodetic survey)	0	6d	12d
Blank	12	13.75 ± 2.32	13.13 ± 2.58	14.16 ± 1.95	13.12 ± 2.56
Model	12	14.35 ± 1.71	13.48 ± 2.79	7.13 ± 1.33*	3.21 ± 1.45*
EA-1	12	14.13 ± 1.78	13.78 ± 2.31	10.39 ± 1.98^#^	10.23 ± 1.59^#^
EA-2	12	13.78 ± 2.32	14.49 ± 1.88	13.46 ± 2.23	13.49 ± 2.82

* *P* < 0.05; Compared with the model group.

^#^ *P* < 0.05.

Compared with the blank control group.

**Table 2:** Effect of electroacupuncture on latency (PWL) of heat-shrinkage group in rats ($\bar{x}\pm s$).

Group	n	PWL(s)			
		base line (in geodetic survey)	0	6d	12d
Blank	12	16.08 ± 1.02	16.65 ± 1.32	16.12 ± 1.35	15.68 ± 1.41
Model	12	16.18 ± 1.18	15.92 ± 1.38	9.65 ± 1.18*	3.55 ± 0.95*
EA-1	12	15.95 ± 1.39	15.88 ± 1.33	8.72 ± 1.10^#^	11.03 ± 1.44^#^
EA-2	12	16.07 ± 1.35	16.38 ± 1.28	16.29 ± 1.44	16.28 ± 1.35

* *P* < 0.05; Compared with the model group.

^#^ *P* < 0.05.

Compared with the blank control group.

### 4.2 Comparison of the levels of GFAP, p-GSK-3β in rats in each group

**Figure 3:**
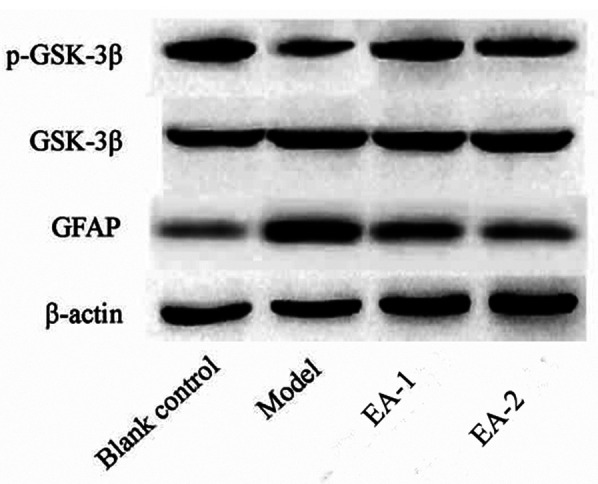
Comparison of the levels of GFAP, p-GSK-3β in rats of each group.

On the 12th postoperative day, in the model group compared with the blank control group, the content of spinal p-GSK-3β (serine9, inactivated GSK-3β) was significantly reduced, and the content of GFAP was significantly increased, and the differences were statistically significant (all *P* < 0.05), and in the electro-acupuncture-1 group compared with the sham electro-acupuncture group, the content of spinal p-GSK-3β was significantly higher, and on the contrary, the content of GFAP was significantly reduced, and the differences were all statistically significant (all *P* < 0.05), and the difference between electro-acupuncture-2 and blank control groups was not statistically significant (all *P* < 0.05). were statistically significant (all *P* < 0.05), and the difference between the spinal cord p-GSK-3β and GFAP contents in the electroacupuncture-2 group compared with the blank control group was not statistically significant (all *P* > 0.05). In addition, the content of GSK-3β in each group was not affected by drug administration (all *P* > 0.05), as shown in Table 3 and Figure 3.

**Table 3:** Effects of electroacupuncture on the expression of GFAP, p-GSK-3β in rats.

Group	p-GSK-3β	GFAP	GSK-3β
Blank	1.08 ± 0.05	1.02 ± 0.03	1.03 ± 0.02
Model	0.53 ± 0.02	2.65 ± 0.28*	1.04 ± 0.01
EA-1	1.15 ± 0.11	1.79 ± 0.18^#^	1.05 ± 0.02
EA-2	1.13 ± 0.09	1.12 ± 0.11	1.03 ± 0.03

* *P* < 0.05; Compared with the model group.

^#^ *P* < 0.05.

Compared with the blank control group.

### 4.3 Comparison of Il-1β, Il-6 and TNF-α contents in spinal cord of rats in each group

**Figure 4:**
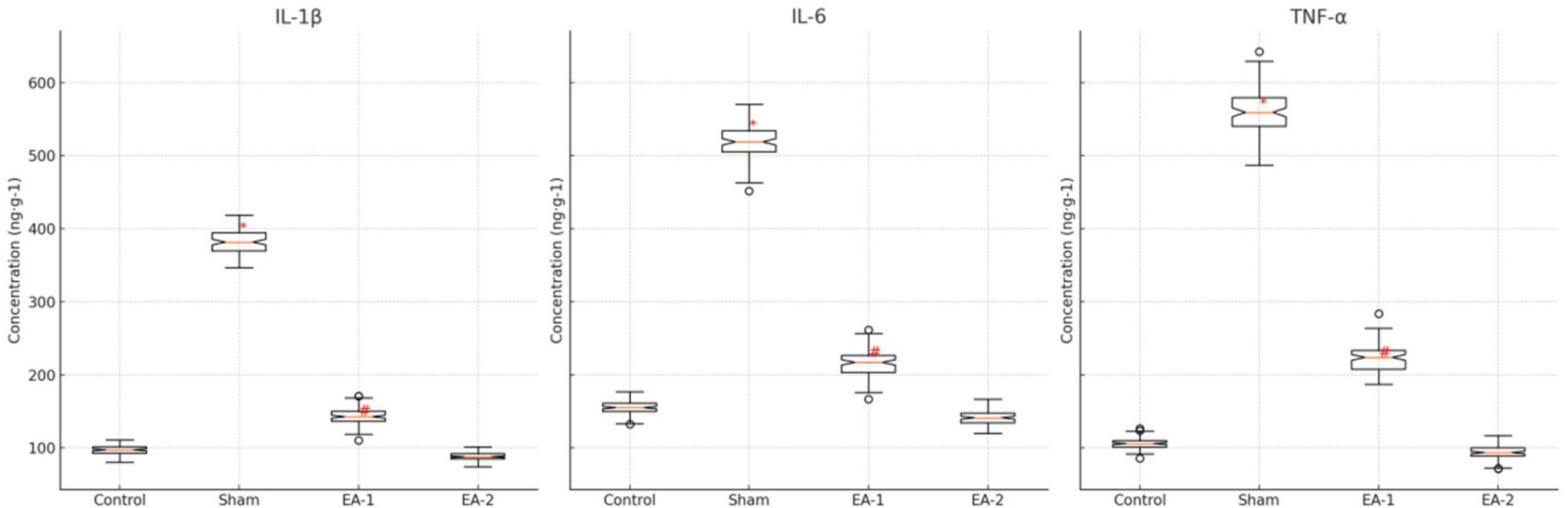
Comparison of spinal cord IL-1β, IL-6, and TNF-α content in rats in each group.

On the 12th postoperative day, the levels of spinal cord IL-1β, IL-6, and TNF-α were significantly higher in the model group compared with the blank control group, and the differences were statistically significant (all *P* < 0.05), while the levels of spinal cord IL-1, IL-6, and TNF-α were significantly lower in the electroacupuncture-1 group compared with the model group, and the differences were statistically significant (all *P* < 0.05), and the differences in the above indexes were not statistically significant (all *P* > 0.05) when comparing the electroacupuncture-2 group with the blank control group, see Table 4, Figure 4. group, none of the differences in the above indexes were statistically significant (all *P* > 0.05), see Table 4, Figure 4.

**Table 4:** Effects of electroacupuncture on IL-1β, IL-6 and TNF-α levels in rats.

Group	IL-1β (ng-g-1)	IL-6 (ng-g-1)	TNF-α (ng-g-1)
Blank	96.77 ± 6.35	154.92 ± 8.22	106.22 ± 7.88
Model	381.26 ± 15.65*	523.35 ± 23.56*	553.26 ± 32.26*
EA-1	143.56 ± 12.02^#^	218.26 ± 18.22^#^	222.36 ± 19.35^#^
EA-2	89.16 ± 5.48	142.32 ± 9.85	91.65 ± 9.95

* *P* < 0.05; Compared with the model group.

^#^ *P* < 0.05.

Compared with the blank control group.

### 4.4 Comparison and analysis of pathological sections of rat tibias

**Figure 5:**
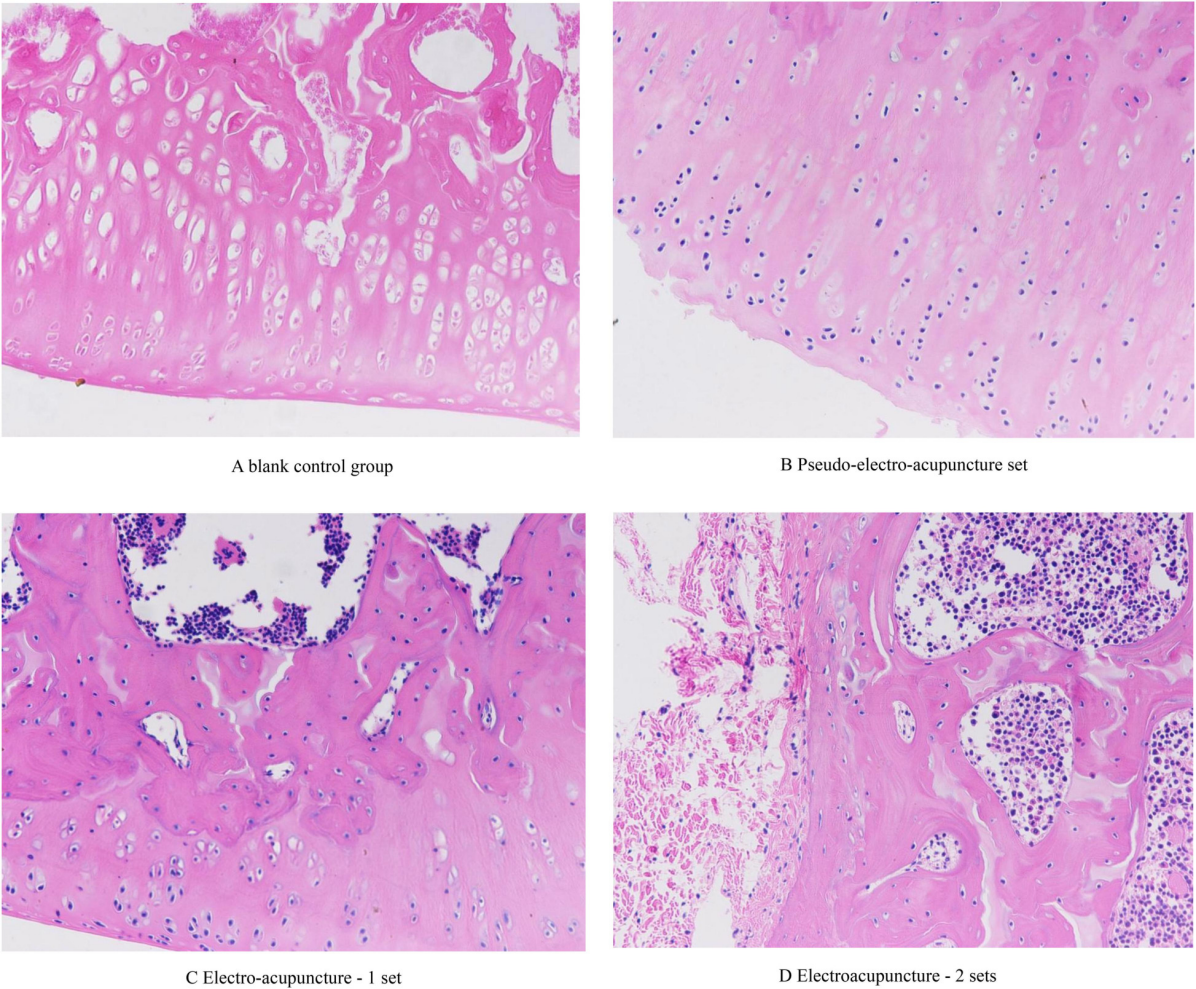
Histopathological staining of rat tibiae in various groups (HE staining, ×200x). **(A)** blank control group. **(B)** Pseudo-electro-acupuncture set. **(C)** Electro-acupuncture—1 set. **(D)** Electroacupuncture—2 sets.

The HE stained section of the blank control group under 200× light microscope showed that the structure of the new bone tissue was clear, the chondrocyte columns were arranged in an orderly manner, and no osteoclasts were seen to proliferate in it, see Figure 5A. The HE stained section of the pseudo-electro-acupuncture group under 200× light microscope showed that the texture of the new bone tissue was ambiguous, the chondrocyte columns were damaged, and a large number of osteoclasts proliferated in the resorption depressions, see Figure 5B. In the HE stained section of group 1, a large number of cancer cells were seen infiltrating between the excessive bone trabeculae under 200× light microscope, indicating that electroacupuncture intervention did not change the pathological state of the local tissues of the lesion, see Figure 5C. A large number of cancer cells were seen infiltrating in the medullary cavity in the HE stained section of group 2, as shown in the light microscope under 200× light microscope. This shows that electroacupuncture did not change the pathological state of the local tissues of the lesion, see Figure 5D.

### 4.5 Comparison of peripheral blood monocyte counts among groups

As shown in Table 5, the model group exhibited a significant increase in peripheral blood monocyte counts compared to the blank control group (*P* < 0.001), suggesting a systemic inflammatory response associated with tumor progression. Both electroacupuncture groups (EA-1 and EA-2) demonstrated reduced monocyte levels relative to the model group (*P* < 0.01), with EA-2 producing a more pronounced suppression (*P* < 0.05 vs. EA-1).

**Table 5:** Effects of electroacupuncture on peripheral blood monocyte counts in rats (×10^9^/L).

Group	Peripheral monocyte count
Blank	0.311 ± 0.015
Model	0.392 ± 0.020*
EA-1	0.348 ± 0.018^#^
EA-2	0.502 ± 0.026

* *P* < 0.05; Compared with the model group.

^#^ *P* < 0.05.

Compared with the blank control group.

### 4.6 Immunofluorescence co-localization of tumor-infiltrating immune markers and astrocytic/p-GSK-3β in the spinal cord

**Figure 6:**
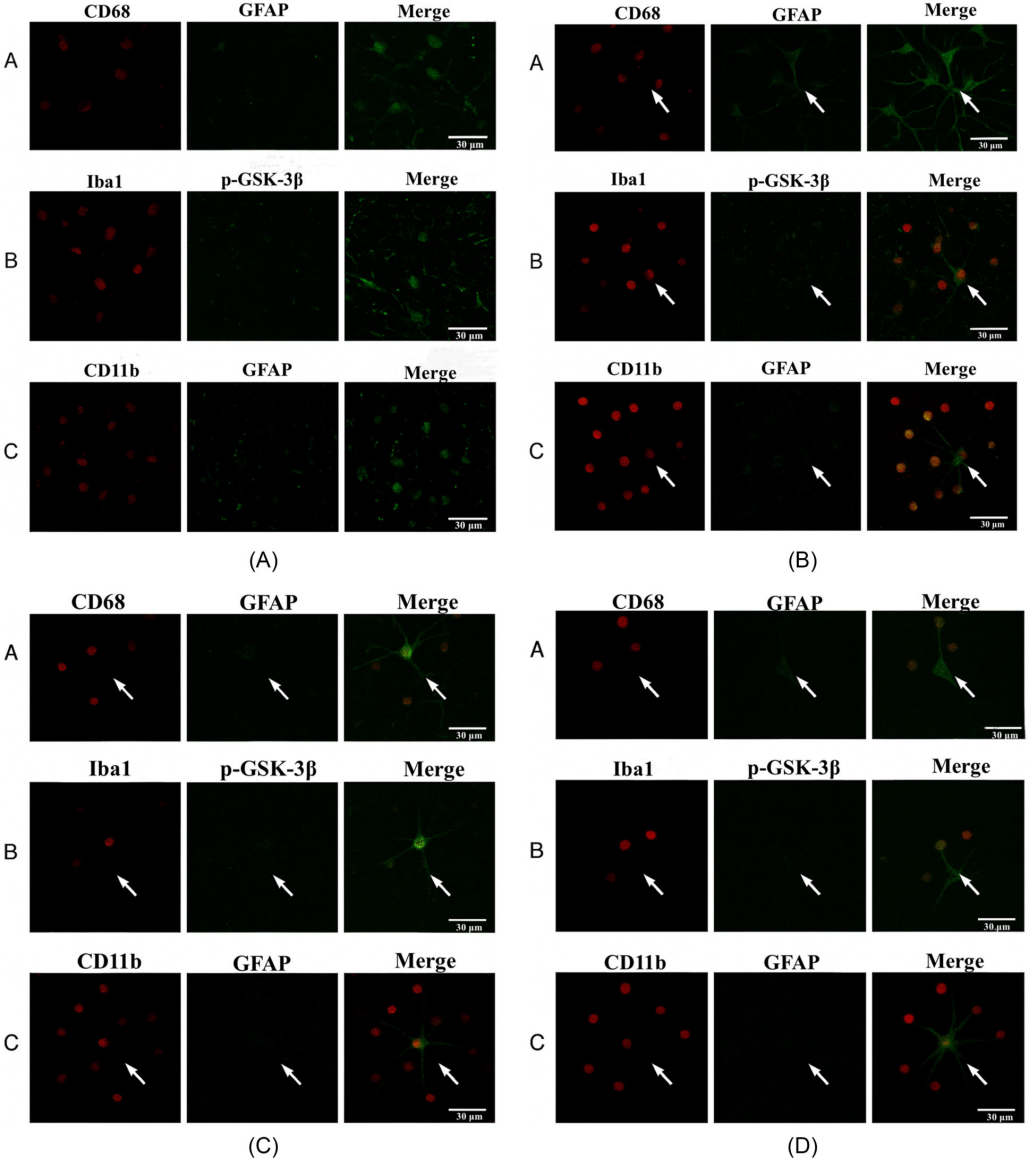
Immunofluorescence Co-localization of tumor-infiltrating immune cell markers with astrocyte and p-GSK-3β in the spinal cord across experimental groups. **(A)** Blank group CD68 + GFAP, Iba1 + p-GSK-3, CD11b + GFAP. Minimal overlap, weak co-localization. **(B)** Sham group -Strong yellow merged signals indicate intense immune-glial interaction. **(C)** EA-1 group-Reduced co-localization, suggesting partial suppression of inflammatory signaling. **(D)** EA-2 group-Sparse and dim merged signals indicate effective suppression of central immune activation.

To further investigate the role of electroacupuncture (EA) in modulating central immune inflammation in cancer-induced bone pain, double-label immunofluorescence staining was performed in all groups to detect co-expression of CD68 + GFAP, Iba1 + p-GSK-3, and CD11b + GFAP in spinal cord tissue.In the Blank group, all three marker pairs showed weak or negligible overlap between red and green channels, suggesting minimal basal activation between immune and glial cells.In the Sham group, co-localization signals were significantly enhanced for all marker pairs, particularly for CD68 + GFAP and CD11b + GFAP. Dense yellow merged signals suggest pronounced astrocyte activation and inflammatory cell infiltration. In the EA-1 group, co-localization intensity was markedly reduced compared to the Sham group. The merged yellow signals were less numerous and dimmer, indicating that EA suppressed abnormal activation between immune and glial cells. In the EA-2 group, co-localization signals were further diminished, suggesting that EA-2 may exert superior effects in suppressing neuroinflammation. see Figure 6, Table 6.

**Table 6:** Effects of electroacupuncture on Pearson's colocalization coefficient in each group.

Group	CD68 + GFAP (PCC)	CD11b + GFAP (PCC)	Iba1 + p-GSK-3 (PCC)
Blank	0.12 ± 0.03	0.10 ± 0.02	0.11 ± 0.04
Sham	0.68 ± 0.05	0.71 ± 0.04	0.65 ± 0.06
EA-1	0.36 ± 0.04	0.33 ± 0.03	0.35 ± 0.05
EA-2	0.28 ± 0.06	0.30 ± 0.05	0.27 ± 0.04

### 4.7 Effects of electroacupuncture on bone matrix and osteoclast markers

**Figure 7:**
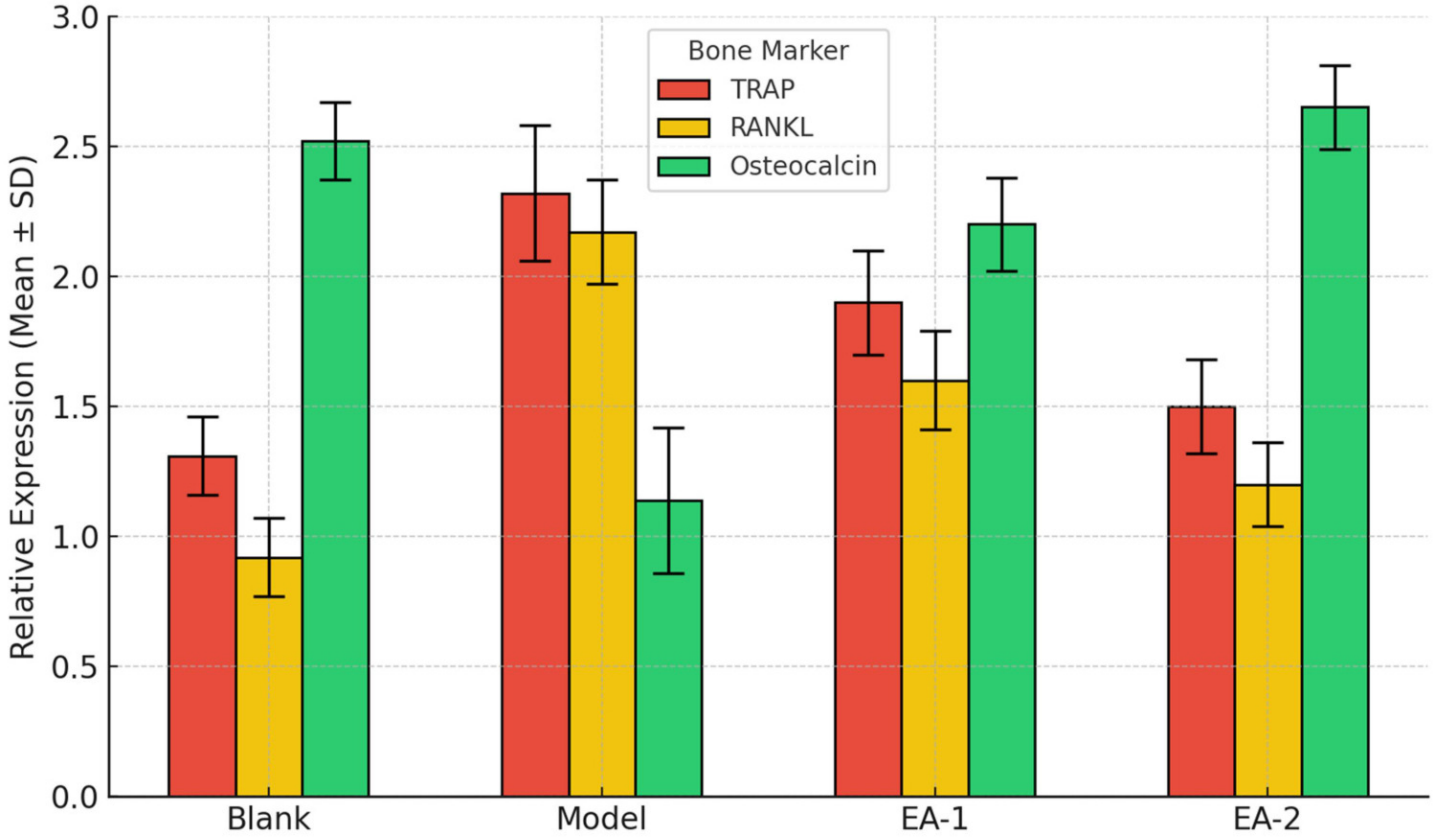
Bar graphs showing the relative expression levels of TRAP, RANKL, and osteocalcin in each group. Data are presented as Mean ± SD (*n* = 8 per group). EA-2 significantly outperformed EA-1 and the model group.

To evaluate the impact of electroacupuncture on bone resorption and formation, we assessed the expression levels of key markers: TRAP and RANKL (associated with osteoclast activity) and osteocalcin (a marker of bone formation). As shown in Figure 7 and Table 5, the model group exhibited significantly increased TRAP and RANKL levels and reduced osteocalcin expression compared to the blank group, indicating enhanced bone resorption and impaired bone formation. Electroacupuncture intervention, especially in the EA-2 group, significantly suppressed TRAP and RANKL expression while enhancing osteocalcin levels, suggesting its superior efficacy in reversing bone metabolic imbalance. see Figure 7, Table 7.

**Table 7:** Comparison of bone matrix integrity and osteoclast activity in different groups of rats.

Group	TRAP (Mean ± SD)	RANKL (Mean ± SD)	Osteocalcin
Blank	1.31 ± 0.15	0.92 ± 0.15	2.52 ± 0.15
Model	2.32 ± 0.26*	2.17 ± 0.20*	1.14 ± 0.28*
EA-1	1.90 ± 0.20^#^	1.60 ± 0.19^#^	2.20 ± 0.18^#^
EA-2	1.50 ± 0.18	1.20 ± 0.16	2.65 ± 0.16

* *P* < 0.05; Compared with the model group.

^#^ *P* < 0.05.

Compared with the blank control group.

## 5 Discussion

Cancer remains a leading cause of mortality worldwide, imposing a significant socioeconomic burden. Recent statistics indicate that China is projected to experience approximately 4.82 million new cancer cases and 3.21 million cancer-related deaths each year (12). Notably, 90% of cancer fatalities are attributed to tumor metastasis rather than the primary tumor itself, with bone being a frequent site of metastasis (13). About 75%–90% of patients with metastases endure moderate to severe pain, and over 80% of cancer pain cases are due to bone pain resulting from tumor spread. Bone cancer pain encompasses elements of acute, inflammatory, and neuropathic pain, leading to osteolysis, spinal cord compression, and considerable pain that profoundly disrupts patients' daily lives (1).

It is important to clarify that the current study employed a localized bone cancer pain model induced by intra-tibial injection of MRMT-1 rat mammary carcinoma cells. This model has been widely used to replicate the pathophysiological and behavioral features of cancer-induced bone pain (CIBP), including tumor cell proliferation within the bone marrow cavity, osteolytic destruction, robust glial activation, and nociceptive hypersensitivity. However, this approach does not model systemic metastatic dissemination and therefore does not represent clinical stage IV cancer. To address this, we have revised the terminology in the manuscript to refer to a “bone metastatic cancer model” or “bone cancer pain model” instead of “stage IV cancer”, to avoid misinterpretation.Furthermore, in our study, cancer cells were confined to the medullary cavity of the right tibia. We did not intentionally induce or evaluate metastasis to other organs. Although post-mortem examinations revealed no visible abnormalities in major organs such as the liver, lung, or spleen, detailed histopathological or imaging analysis of systemic spread was beyond the scope of the present investigation. Future studies utilizing *in vivo* bioluminescence imaging, PET/CT, or organ-specific histology would be valuable to assess metastatic potential and spillover effects more comprehensively.Regarding the anatomical specificity of electroacupuncture (EA), the stimulation was applied at bilateral Zusanli (ST36) and Kunlun (BL60), which are located distal to the tumor-inoculated tibial site. The stimulation parameters (2/100 Hz alternating frequency at 0.5–2 mA) were designed to engage systemic analgesic pathways, including the activation of descending inhibitory circuits and modulation of glial signaling. The current intensity and location were not expected to exert direct mechanical or electrical influence on the tibial lesion site. Consistently, histological analysis revealed no changes in local tumor infiltration or bone integrity between EA and sham groups, indicating that EA's analgesic effects were mediated centrally, rather than through local modulation of the shin bone or tumor microenvironment. In this study, electroacupuncture interventions were initiated on day six post-tumor cell inoculation rather than concurrently. This timing was based on the well-established progression of cancer-induced bone pain in rat models, wherein significant nociceptive behaviors typically emerge between days 5 and 7 after tumor cell implantation. Initiating treatment on day six ensured that measurable pain-related behavioral changes—such as reductions in paw withdrawal threshold (PWT) and paw withdrawal latency (PWL)—had already developed, thus providing a reliable baseline to evaluate the therapeutic efficacy of electroacupuncture. Administering intervention prior to pain establishment would reflect a prophylactic rather than therapeutic model, which does not align with clinical treatment scenarios where patients often present with established pain. Furthermore, we observed that on day six, PWL values in the EA-2 group were marginally higher than those in the EA-1 group. This transient elevation may be attributed to individual variation in pain progression or to a stress-induced analgesic response triggered by handling or the anticipation of stimulation, as neither group had received active intervention by that point. It is also possible that the higher stimulation intensity planned for the EA-2 group (2 mA for 15 min) induced early physiological changes due to animal conditioning. However, this discrepancy was not sustained—by day twelve, the EA-1 group exhibited superior analgesic outcomes in both PWT and PWL, supporting the effectiveness of moderate-intensity, consistent electroacupuncture stimulation over intermittent high-intensity protocols. Electroacupuncture is known to activate Aδ and C nerve fibers at specific acupoints, triggering signal transmission to the spinal cord and midbrain structures like PAG and RVM, thus engaging endogenous opioid and monoaminergic systems. Additionally, several studies suggest that EA may increase local blood perfusion and spinal cord microcirculation, which can modulate pain signaling (14). In contrast, sham electroacupuncture lacks electrical stimulation, providing only minor sensory input that does not activate neurovascular responses, thereby serving as a placebo-like control. The primary mechanism of bone metastasis in cancer cells involves their entry into the bloodstream, where they evade programmed cell death to ensure survival. This resistance is primarily mediated through the overexpression of tropomyosin receptor kinase B (TrkB) and the activation of the PI3K-AKT pathway. The process by which cancer cells invade bone tissue is complex, primarily driven by the CXCL12-CXC chemokine receptor axis. Once within the bone, cancer cells proliferate rapidly, forming tumors that damage the bone structure (15). These tumors attract macrophages, T cells, neutrophils, mast cells, and other inflammatory cells, which release mediators such as NGF, IL-6, and bradykinin, sensitizing bone-innervated sensory nerve endings and triggering nociceptive responses (16). Electroacupuncture, a traditional Chinese medicine technique, has demonstrated efficacy in alleviating pathological and inflammatory pain, although its effects on bone cancer pain, particularly through mechanisms involving GSK-3β downregulation, astrocyte inhibition, and inflammatory reduction, are less reported. This study, using a rat bone cancer pain model, found that electroacupuncture increased PWT and PWL expression levels and reduced levels of spinal p-GSK-3β, GFAP, IL-1, IL-6, and TNF-α. HE staining revealed that the tibial bone tissue in the control group had a clear structure with orderly chondrocyte columns, lacking osteoclast proliferation. In contrast, sham and electroacupuncture groups exhibited significant cancer cell infiltration within trabecular spaces, disrupted new bone tissue, and damaged chondrocyte columns with extensive osteoclast proliferation. These findings suggest that electroacupuncture provides short-term pain relief in bone cancer pain but does not inhibit cancer cell invasion into bone tissue.In addition to modulating central glial activity and cytokine expression, our results showed that electroacupuncture also exerted peripheral immunomodulatory effects. Tumor-bearing rats displayed elevated peripheral monocyte counts, consistent with previous reports of systemic inflammation in cancer-induced bone pain (17). EA treatment significantly suppressed these monocyte levels, particularly in the EA-2 group, suggesting that electroacupuncture may mitigate not only spinal but also systemic immune responses. The reduction in circulating monocytes may reflect decreased mobilization or activation of monocyte-lineage cells, which are known to contribute to neuroinflammation via spinal infiltration. These findings support a dual mechanism of EA, involving both central and peripheral pathways of immunoregulation.

To investigate the mechanisms underlying electroacupuncture's effects, various studies have highlighted that glial cells play a significant role in pain development. Known as glial cells, these non-neuronal cells in the nervous system comprise microglia, astrocytes, and oligodendrocytes within the central nervous system (CNS) (18). Astrocytes are particularly crucial, as they are deeply involved in neuroimmune modulation and neuronal signal transmission, contributing to central sensitization and sustaining pain (19). Activation of astrocytes in the spinal dorsal horn is reflected by increased GFAP expression. Activated astrocytes at the spinal level release several pro-inflammatory cytokines, such as TNF-α, IL-1β, and TGF-β (20, 21), which promote neurotransmitter release and further activate pain signaling pathways (22). Recent research has also demonstrated that chemokines expressed in the CNS interact with spinal glia, contributing to pain pathogenesis (15). Our findings align with previous studies, leading us to hypothesize that electroacupuncture's analgesic effects may stem from inhibiting astrocyte activation and reducing pro-inflammatory cytokine expression, thus dampening pain transmission. This study further reveals that electroacupuncture attenuates inflammation. GSK-3β has been reported as a critical modulator in inflammatory response pathways, as its expression in glial cells elevates pro-inflammatory cytokines upon activation. Conversely, inhibiting GSK-3β with the small molecule SB216763 reduces the release of IL-1β and TNF-α from glial cells (23). Inhibition of GSK-3β or application of GSK-3β-siRNA increases anti-inflammatory cytokine IL-1R production in response to lipopolysaccharides (LPS) (24). GSK-3β plays an essential role in immune responses in the CNS, modulating the balance between pro- and anti-inflammatory effects. Its activity mainly relies on phosphorylation at the Ser9 site, with lower p-GSK-3β (Ser9) levels leading to activation of GSK-3β. Additionally, GSK-3β directly phosphorylates the Ser468 site of the NF-κB subunit, regulating NF-κB's baseline activity (25). Furthermore, GSK-3β disrupts the IκB signaling, which retains NF-κB in the cytoplasm, thereby lowering its DNA-binding and transcriptional activity to mitigate inflammation (26). We propose that electroacupuncture may reduce inflammation by inhibiting spinal GSK-3β activity.In this study, we confirmed that electroacupuncture (EA) significantly attenuates bone cancer pain, accompanied by reduced spinal astrocyte activation (GFAP), elevated phosphorylated GSK-3 (p-GSK-3) levels, and suppression of inflammatory cytokines. To further investigate the underlying mechanism and address the reviewer's concern regarding immune-glial interactions, we performed double immunofluorescence staining to examine co-localization of tumor-infiltrating immune markers with astrocytic and signaling components in the spinal cord.In the sham group, merged fluorescence signals between CD68 and GFAP, CD11b and GFAP, and Iba1 and p-GSK-3 were significantly enhanced, indicating close spatial proximity between activated macrophages/microglia and astrocytes under bone cancer conditions. This pattern of co-localization was accompanied by increased expression of GFAP and decreased levels of p-GSK-3, suggesting that immune infiltration may contribute to astrocytic activation and downstream dephosphorylation of GSK-3 through local paracrine inflammatory signaling.In contrast, EA treatment—particularly in the EA-1 group—markedly reduced co-localization signals between CD68/GFAP and Iba1/p-GSK-3, indicating that electroacupuncture disrupts immune-glial spatial interactions and the associated pro-inflammatory signaling cascades. The EA-2 group also showed reduced co-expression, though to a lesser extent. These findings, together with the biochemical data, support a model wherein tumor-infiltrating CD68+, CD11b+, and Iba1+ immune cells promote astrocytic reactivity, which facilitates GSK-3 dephosphorylation and contributes to neuroinflammation and central sensitization. Electroacupuncture appears to block this pathway by preserving GSK-3 phosphorylation and reducing astrocyte-mediated inflammatory responses.

In addition to modulating glial activation and inflammatory cytokine production, electroacupuncture (EA) has been increasingly recognized to influence multiple neurobiological pathways involved in pain regulation. One such mechanism is the engagement of descending pain inhibitory systems. EA stimulation has been shown to activate neurons in the periaqueductal gray (PAG) and rostral ventromedial medulla (RVM), leading to enhanced endogenous opioid peptide release and subsequent suppression of nociceptive transmission at the spinal level. This process involves μ-opioid receptors, serotonergic and noradrenergic pathways, all of which are integral to central pain inhibition.

Moreover, EA may alter the excitability of primary afferent neurons and spinal interneurons by modulating ion channel expression, such as voltage-gated sodium channels (Nav1.7, Nav1.8) and transient receptor potential vanilloid 1 (TRPV1). These ion channels are crucial mediators of nociceptive signal initiation and amplification in cancer-induced bone pain. The suppression of TRPV1 expression, in particular, has been linked to decreased thermal hyperalgesia.Neuroimmune interactions also play a significant role in the pain process. Emerging evidence suggests that EA may inhibit microglial polarization toward the M1 pro-inflammatory phenotype, thereby reducing the release of TNF-α, IL-1β, and IL-6. Simultaneously, it may promote M2 anti-inflammatory polarization, contributing to resolution of neuroinflammation. Furthermore, EA has been reported to suppress the activation of the NLRP3 inflammasome and the TLR4/NF-κB pathway, which are known to drive neuropathic pain states in cancer models.Finally, EA may influence epigenetic and transcriptomic regulation within the spinal cord and supraspinal structures. Studies have demonstrated changes in microRNA expression profiles and histone acetylation patterns following EA treatment, which can affect the expression of pain-related genes such as BDNF, c-fos, and CREB (27, 28). These epigenetic changes may underlie the sustained analgesic effects of repeated EA sessions, providing a new perspective on its long-term efficacy (29–31).

To conclude, electroacupuncture demonstrated an ability to reduce the pain threshold in a rat model of bone cancer pain, likely through mechanisms involving down-regulation of GSK-3β activity, inhibition of astrocyte activation, and mitigation of inflammatory responses. Electroacupuncture combines traditional acupuncture with electrical currents and has shown promise in clinical analgesia. Nonetheless, due to limitations in current research models and techniques, ex vivo and mechanistic studies on electroacupuncture remain insufficient. Given the strong link between acupuncture efficacy and neurophysiological mechanisms, future studies should employ advanced neurophysiological approaches to further elucidate the central mechanisms underlying electroacupuncture-induced analgesia. For instance, *in vivo* electrophysiological recording of dorsal horn neurons can directly measure neuronal excitability changes; optogenetic manipulation of spinal glial cells allows for precise cell-type-specific functional interrogation; and calcium imaging enables real-time monitoring of neuronal activity dynamics. These techniques will help clarify the neurobiological basis of electroacupuncture and provide empirical support for optimizing its clinical application.

## Data Availability

The original contributions presented in the study are included in the article/Supplementary Material, further inquiries can be directed to the corresponding author/s.
